# 2577. Comparing Omadacycline with Existing Antibiotics in the treatment of Acute Bacterial Infections: A Systematic Review

**DOI:** 10.1093/ofid/ofad500.2193

**Published:** 2023-11-27

**Authors:** Stephanie P Fabara, Akankcha Alok, Sripal Padam, Robert Yancey

**Affiliations:** University of Central Florida, Gainesville, Florida; University of Central Florida, Gainesville, Florida; University of Central Florida, Gainesville, Florida; University of Central Florida, Gainesville, Florida

## Abstract

**Background:**

Omadacycline (OMC), the first aminomethylcycline antibiotic, is a semisynthetic tetracycline derivative approved for community-acquired bacterial pneumonia (CABP) and acute bacterial soft skin infection (ABSSI), with a broad range of activity against Gram-positive and Gram-negative aerobes, anaerobes and atypical bacteria, including drug-resistant strains. It is currently under development for the treatment of cystitis and acute pyelonephritis (AP).

**Methods:**

This systematic review of literature followed the Preferred Reporting Items for Systematic Reviews and Meta-Analysis (PRISMA) guidelines. Abstracts were searched using the term: “omadacycline”. The electronic research literature databases included the Cochrane Library, MedLine, and clinicaltrials.gov from February to April 2023. Selection of studies by PRISMA flowchart is shown in figure 1. Characteristics of endpoints for each pathology are mentioned in table 1.

**Figure:**
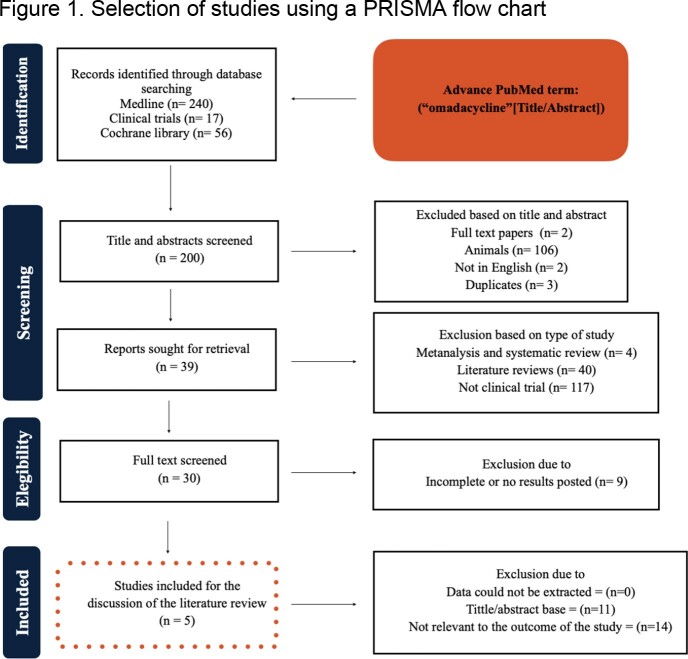

**Table 1.**
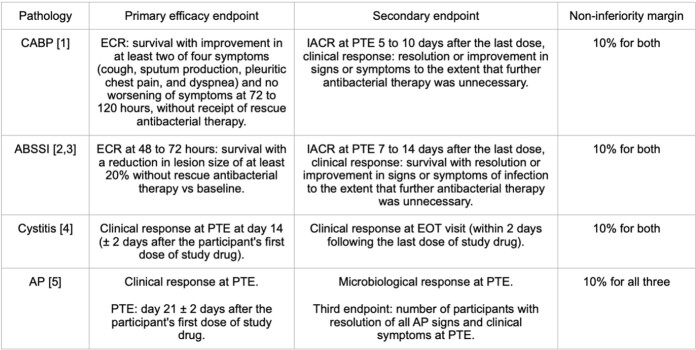
Endpoints for each disease. CABP (Community-acquired Pneumonia), ECR (Early clinical response), Investigator-assessed clinical response (IACR), Post treatment evaluation (PTE), ABSSI (Acute bacterial soft skin infection), EOT, (End of treatment), AP (Acute pyelonephritis).

**Results:**

OMC was found to be noninferior to existing antibiotics in all reviewed studies. The most common side effects of OMC were nausea, vomiting and diarrhea, which were infrequent, mild and easily treatable. The only limitation of these studies was proving the efficacy of OMC by non-inferiority. Bias was assessed using the Cochrane collaboration's tool risk assessment of the clinical trials (Figure 2). Characteristics and outcomes of each study are mentioned in table 2.

**Figure:**
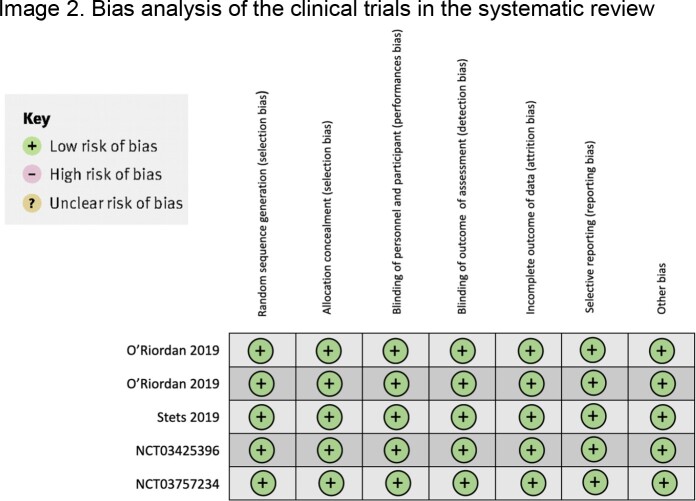

**Table 2.**
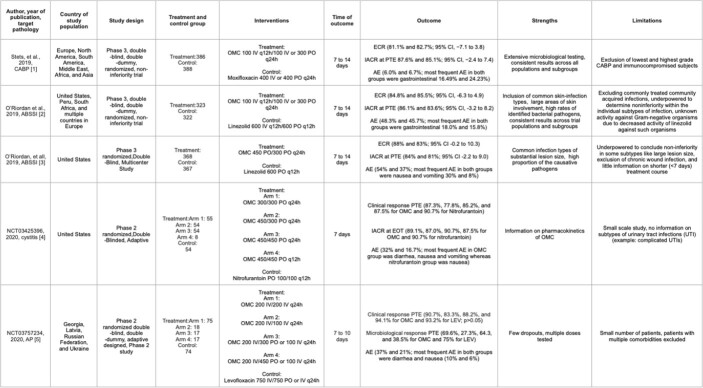
Characteristics, outcomes and limitations of selected studies. CABP (Community-acquired Pneumonia), OMC (Omadacycline), IV (Intravenous), ECR (Early clinical response, IACR (Investigator-assessed clinical response), PTE (Post-treatment evaluation), AE (Adverse effect), ABSSI (Acute bacterial soft skin infection), EOT (End of treatment), AP (Acute pyelonephritis), and LEV (Levofloxacin).

References

**Figure:**
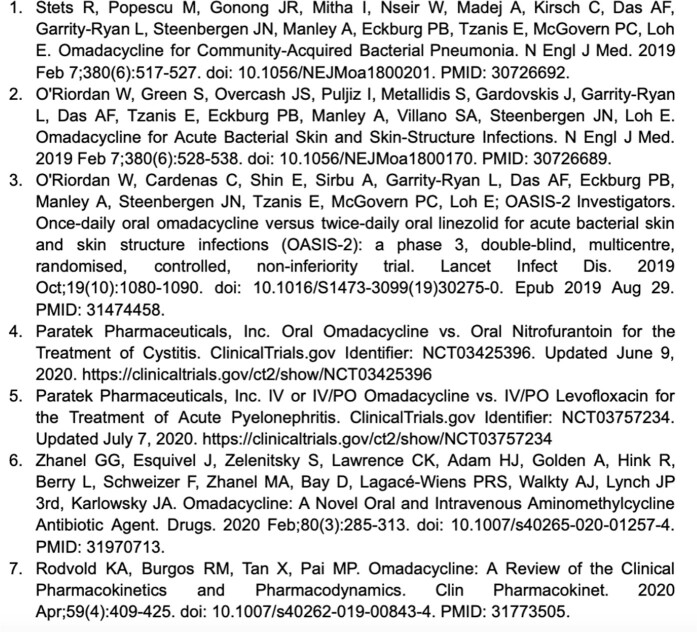

**Conclusion:**

Most studies showed that OMC was well tolerated, with decreased frequency of dosing, availability of both oral and intravenous options, and with equal if not better efficacy than the existing drugs. OMC has activity against common tetracycline resistance mechanisms such as efflux pumps and ribosomal protection proteins. It is excreted unchanged, having low potential for drug-drug interaction and requires no dose adjustment for age, sex, or hepatic and renal impairment. Further large scale studies are encouraged to prove OMC as an emerging antibiotic of choice for several bacterial infections. Overall, OMC appears to be a promising drug with a good safety profile. With the help of this systematic review, we aim to encourage the use of OMC for cystitis and pyelonephritis, along with continued use for CABP and ABSSI.

**Disclosures:**

**All Authors**: No reported disclosures

